# Neurosarcoidosis Mimicking Normal Pressure Hydrocephalus

**DOI:** 10.7759/cureus.40281

**Published:** 2023-06-12

**Authors:** Aida Kafai Golahmadi, Claudia L Craven, Laurence D Watkins

**Affiliations:** 1 Neurosurgery, National Hospital for Neurology and Neurosurgery, London, GBR

**Keywords:** obstructive hydrocephalus, normal pressure hydrocephalus, sarcoidosis, hydrocephalus, neurosarcoidosis

## Abstract

Two female patients, aged 46 and 51, were referred to the National Hospital for Neurology and Neurosurgery with symptoms resembling normal pressure hydrocephalus (NPH) and with ventriculomegaly on the MRI.

Both had a definite diagnosis of neurosarcoidosis (NS) on biopsy, and they underwent the medical and surgical management reserved for NPH. At follow-up, their presenting symptoms had resolved and they had clinically improved. Neurosarcoidosis can mimic NPH, and it should be excluded in patients presenting with NPH.

## Introduction

Neurosarcoidosis is a rare condition, diagnosed in 5-13% of patients with systemic sarcoidosis; however, it has a high mortality rate, and it can affect any part of the central and peripheral nervous system [1-4]. In 5-7% of cases, it can lead to communicating and non-communicating hydrocephalus [4-6], and the latter is a potentially lethal disease with serious long-term complications as a result of chronic pachymeningitis of the arachnoid granulations or due to inflammation of the choroid plexus [6,7].

Idiopathic normal pressure hydrocephalus (iNPH) refers to a ventricular enlargement with normal opening pressures on lumbar puncture, and it presents with the classic triad of dementia, gait disturbance, and urinary incontinence [5].

The presence of neurological manifestations, which usually occur within two years from diagnosis of sarcoidosis, should prompt a work-up for neurosarcoidosis [3,7].

Lumbar puncture (LP) and cerebrospinal fluid (CSF) analysis should always be considered, despite their low diagnostic yield, as elevated immunoglobulin (Ig)G index, oligoclonal bands, and high levels of elevated angiotensin enzyme can support the diagnosis of neurosarcoidosis [7].

In addition, elevated protein levels and cerebrospinal fluid opening pressure, along with lymphocytic pleocytosis, predominantly mononuclear cells, are characteristic of normal pressure hydrocephalus [5-7].

## Case presentation

We report two cases with definite neurosarcoidosis, confirmed on tissue biopsy, who were referred to the National Hospital for Neurology and Neurosurgery for hydrocephalus. They presented with one year of cognitive decline and magnetic gait, mimicking normal pressure hydrocephalus. They both had a previous diagnosis of systemic sarcoidosis, with lesions in their lungs and elevated serum angiotensin-converting enzyme (ACE) levels (>40 micrograms/L).

Case 1

A 46-year-old woman with a 13-month history of mobility decline. Her family had reported that she was more apathetic over the last six months. On examination, her gait was magnetic, and it took her 52 steps over 40 seconds to walk 10 meters. She was also noted to have right-sided facial nerve palsy. All other cranial nerves were intact. She underwent neuropsychological tests, which were standard at that time, including the Wechsler Adult Memory Scale-Revised Edition (WAIS-R). The WAIS-R test found her to have a global cognitive deficit, with particularly poor functioning in attention and calculation. The score in each subtest was collected by a neuropsychologist who then gave us a summary report with the average score, which, in this case, was 98.

Her CSF analysis from the lumbar puncture test showed pleocytosis and elevated proteins.

**Figure 1:**
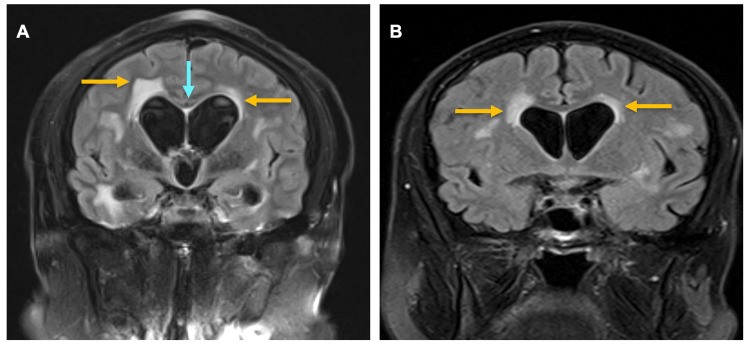
Coronal MRI showing a narrow callosal angle (blue arrow) in Figure 1A and periventricular white matter changes (orange arrow) in Figures 1A and 1B

Her coronal MRI with gadolinium showed a narrow callosal angle (Figure 1A), a hallmark of idiopathic normal pressure hydrocephalus (iNPH), and periventricular white matter changes (Figure 1B). In light of the enlarged ventricles, she underwent 24-hour intracranial pressure (ICP) monitoring, with a median ICP of 3.47 mmHg, which is within the normal range, and a pulse amplitude of 4.35 mmHg. 

Case 2

A 51-year-old woman presented with increased falls and difficulty walking over the last 12 months and some mild disorientation. On examination, she also had a slow magnetic and a broad-based gait. She took 47 steps over 40 seconds to walk 10 meters. Her WAIS-R report described a global cognitive deficit with poor performance in executive functions and an average score of 93.

**Figure 2:**
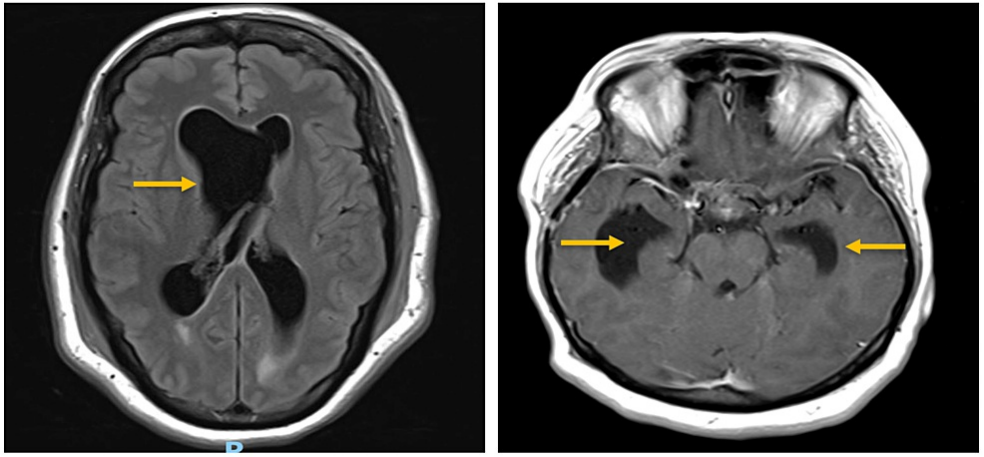
Axial MRI images showing communicating enhancing hydrocephalus (orange arrows)

Her axial MRI with gadolinium showed communicating enhancing hydrocephalus (Figure 2). On the axial MRI, the ventriculomegaly is not symmetrical and one explanation for the laterality of the periventricular enlargement may be that an asymmetrical deposition of neurosarcoid lesions and related inflammation may have led to compartmentalized hydrocephalus.

Cerebrospinal fluid taken from the lumbar puncture showed a mild increase in chronic inflammatory cells in the absence of infection and elevated protein levels. The opening pressure of the LP was 18 mmHg.

Management and outcomes

Both patients received long-term prednisolone for extra-neural sarcoidosis, and they underwent ventriculoperitoneal shunt insertion, with adjustable valves set to 5 cmH2O and anti-siphon devices. There was a propensity for the transient development of slit-like ventricles but without other features of over-drainage. However, the ventricles slowly returned to their normal size when the valve was turned up to 15 cmH2O. At seven months, cognition (WAIS-R) and 10-meter walking speed improved by at least 25% in both patients. For the patient in Case 1, the facial nerve palsy also resolved (owing to the steroid treatment).

## Discussion

Sarcoidosis is a systemic inflammatory disorder that causes granuloma formation in multiple organs, most commonly the lung, and very rarely the nervous system, including the brain. Only half of patients with neural sarcoidosis will be symptomatic and their symptoms depend on which part of their brain or spinal cord is affected [1,8]. The presenting complaints tend to be cranial nerve abnormalities, in particular, facial nerve palsy, followed by confusion, memory loss, headaches, seizures, and weakness in the limbs; however, hydrocephalus is not a common presentation, and therefore, it is important to raise awareness of its potential association with this disease [1,8-12].

In our patients, the cognitive decline could be explained by the involvement of the brain white matter whilst the magnetic gait could be a sign of peripheral nerve involvement.

On clinical examinations, patients may have cervical and submandibular lymphadenopathy and cutaneous lesions; the serum ACE levels will be elevated while the inflammatory markers (CRP and ESR) may be normal or elevated, and the CSF analysis will show lymphodominant pleocytosis and elevated protein [1,2,7,8].

The diagnostic workup usually includes a combination of MRI studies, cerebrospinal fluid analysis, and tissue biopsy. The MRI will show the characteristic ventriculomegaly, periventricular, white matter, and leptomeningeal enhancement, multiple or solitary supra- and infratentorial lesions, as well as intramedullary lesions, and involvement of the cranial nerves [1,2,8,10].

The diagnosis can only be confirmed by a biopsy showing non-caveating granulomas in the affected organs; however, sometimes it is possible to make a diagnosis of probable neurosarcoidosis in a background of systemic sarcoidosis, based on the imaging findings and cerebrospinal fluid analysis [1,2,8].

The mechanisms by which neurosarcoidosis leads to hydrocephalus are not fully understood; however, it has been suggested that the formation of sarcoid granulomas and the resulting scarring in the meninges obstruct the CSF flow while inflammation of the arachnoid villi disrupts its absorption, contributing to an accumulation of fluid in the ventricles via both obstructive, also known as non-communicating hydrocephalus, and communicating hydrocephalus [4-6].

Interestingly, we noted that in patients with normal pressure hydrocephalus secondary to neurosarcoidosis, following shunt placement, the ventricles rapidly decompress and slit-ventricles can occur, unlike in patients with iNPH. Although the cause of this phenomenon is not well-understood, it has been suggested that the fast reduction in pressure within the ventricles due to CSF drainage makes the ventricular walls collapse, causing a reduction in the ventricular size [11].

This was observed in our patients but it resolved spontaneously without treatment, and the ventricles regained their normal size. 

Currently, there are less than 25 cases in the literature describing hydrocephalus as a presenting symptom of sarcoidosis or neurosarcoidosis, with non-communicating hydrocephalus being the most common type, and the majority of them reported partial or complete recovery following the treatment [13]. 

## Conclusions

We report two cases of patients with systemic sarcoidosis who presented with signs and symptoms typical of iNPH and were found to have hydrocephalus secondary to neurosarcoidosis. Hydrocephalus was a reversible cause of the neurological decline in these patients, implying that prompt recognition and prompt interventions are important in this high-mortality disease. Early combined treatment of CSF diversion with a VP shunt, and medical management with systemic corticosteroids, was as effective as in iNPH for symptoms management.

Therefore, we believe that in the context of patients with a definite diagnosis of neurosarcoidosis with hydrocephalus and clinical features of NPH, combination therapy of steroids and VP shunt is effective, and they should both be offered to the patients. Neurosarcoidosis should be considered in patients with systemic sarcoidosis presenting with nonspecific neurological symptoms and hydrocephalus, especially when infectious and malignant causes have been excluded, even in cases where clinical and radiological data are characteristic of iNPH.
